# Draft Genome Sequence of *Bacillus subtilis* QH-1, a Chromium-Reducing Bacterial Strain Isolated in Qinghai Province, China

**DOI:** 10.1128/genomeA.00182-14

**Published:** 2014-03-27

**Authors:** Liang Feng, Teng Ma, Junwen Zhang, Fen Xu, Liu Shi

**Affiliations:** School of Environmental Studies, Hubei Key Laboratory Wetland Evolution & Ecological Restoration, China University of Geosciences, Wuhan, China

## Abstract

*Bacillus subtilis* strain QH-1, a chromium-reducing bacterial strain, was isolated from a soil sample from a chromium-containing slag heap. The draft genome sequence of this bacterium is 4,034,036 bp in length, with a G+C content of 43.71%, and it is predicted to contain 4,082 protein-coding genes.

## GENOME ANNOUNCEMENT

*Bacillus subtilis* is a model Gram-positive bacterium. It is one of the most important groups of industrial microorganisms and is used in several fermentative production processes due to its capacity to secrete large amounts of industrial enzymes (such as amylases and proteases) (1); these enzymes are able to degrade a variety of substrates. *B. subtilis* strain QH-1 was isolated from a soil sample from a chromium-containing slag heap in Qinghai province, China. The strain was shown to be able to reduce hexavalent chromium to the insoluble and less toxic Cr(III).

The draft genome of *B. subtilis* QH-1 was sequenced using Illumina technology. An Illumina standard shotgun library was constructed and sequenced using the Illumina MiSeq platform, which produced 999,334 paired-end reads totaling 501.7 Mb. Potential adapter sequences and low-quality bases were removed using cutadapt (2) and a custom Perl script, respectively. Filtered Illumina reads were assembled using Newbler version 2.8, resulting in an assembly of 4,034,036 bp in 11 contigs. The G+C content of the genome was 43.71%, and the average genome coverage depth was approximately 82.26×.

Annotation was performed using NCBI Prokaryotic Genome Automatic Annotation Pipeline (PGAAP) (3), which predicted a total of 4,189 genes (with 4,082 protein-coding genes and 81 noncoding RNAs). The protein product names were assigned by the PGAAP based on the hits to the NCBI refseq databases, and 3,022 of them were assigned a definite function.

### Nucleotide sequence accession number.

This whole-genome shotgun project has been deposited at GenBank/DDBJ/EMBL under the accession no. AZQS00000000.
